# Diphtheria and Its Associates

**Published:** 1896-04

**Authors:** 


### Book Reviews.

Diphtheria and its Associates. By Lennox Browne, F. R. C. S., Ed., Senior Surgeon to the Central London Throat, Nose and Ear Hospital; late President of the British Laryngological Association; Corresponding Fellow of the American Laryngological Association; Author of The Throat and Nose, and their Diseases, etc., etc. Illustrated by the author. Pp. xii.—272. Price, $5. London: Balliere, Tindall & Cox. Philadelphia: J. B. Lippincott Company. 1895.

Among the many new and valuable books that have appeared recently, there is none that can be said to be more thoroughly up-to-date than the one before us. Nearly all medical monographs are written either from the standpoint of the clinician or of the scientist. Hence, it results that either the clinical observations lack the confirmation of the experimentalist, or the deductions made in the laboratory are not corroborated by clinical application. In no work that has come to our notice of late has this most desirable combination of scientific deduction and clinical application been so fully, ably and clearly made, as in this treatise. The title of the book is a most happy one. It at once gives the reader a clue to the fact that there are forms of mucous inflammation, characterised by a pseudo-membranous formation and caused by micro-örganisms other than those of a diphtheritic character.

This essay, the author states, is based mainly on a course of lectures recently delivered for the purpose of establishing a landmark at an important era in the history of diphtheria, and of emphasising the desirability of assimilating the teachings of bacteriology to the purposes of practical medicine. Mr. Browne has incorporated in his book many original and valuable observations made by himself and recorded in his writings at various times, to which he has added the present teachings of practical bacteriology.

As is quite characteristic of Mr. Browne, the subject is treated in a very careful and systematic manner. He first deals with the history of diphtheria; then he treats of its etiology, pathology, the bacteriological diagnosis of diphtheria and its associates; the clinical diagnosis, record of illustrative cases of diphtheria and its associates; prognosis, treatment—both remedial and operative—laryngo-tracheal diphtheria, croup, and the hygiene and prophylaxis of diphtheria. In addition to these chapters he gives formulæ for remedies and an appendix on the serum treatment of diphtheria.

In his brief résumé of the history of diphtheria, the author takes occasion to pay a just tribute to Sir Morell Mackenzie and the other writers on this subject who have enriched our knowledge of this disease, notwithstanding the fact that its specific cause, the Klebs-Loeffler bacillus, was unknown to them. It was his characteristic rare acumen that led Mackenzie to assert the unity of croup and diphtheria, which bacteriological research finally corroborated. The discovery of the specific organism by Klebs in 1883, and the establishing of the causal relationship of this organism to diphtheria, in 1884, by Loeffler, by making pure cultures and inoculating guinea-pigs and other animals with the disease, marks the crowning epoch in its study and history.

"Logically," the author says, "the etiology of diphtheria might be comprised in a description of its specific organism, with its local manifestations and its systemic effects, and such has been the course pursued by those who are commonly described as scientists." This, however, is to be regarded only as the exciting cause, whereas the study of the predisposing causes, those that favor the development of the germ, is of no less importance. It is this cause that accounts for the mortality of the disease, which is three times greater in rural districts than in urban. The greater mortality of the disease in badly-drained, unsanitary dwellings; in dwellings in close proximity to stables, cess-pools, and the like; in regions of excessive soil-moisture, and the fact that the disease is more prevalent during the seasons of the year that are cold and damp, are owing to the same cause.

Not only does germ infection take place from external sources, but systemic conditions may equally favor the development of germs. This fact, which is often overlooked by writers on this subject, has here, we are happy to say, due prominence. The importance of proper attention to conditions of the nose and throat that necessitate mouth-breathing and that also afford a most excellent culture medium, cannot be overestimated. Such conditions are found mainly in adenoid vegetations or enlarged tonsils. The tonsils, which Virchow so aptly terms "open wounds," owing to their frequently diseased condition, are the parts in which the diphtheritic bacillus most frequently gains foothold. Mr. Browne properly and most forcibly calls attention to the removal of these diseased conditions as a prophylactic. Of 1,000 cases tabulated by him, the exudate was reported to be limited to the tonsillar region in 666 cases. In eight only of the 1,000 were the tonsils not implicated with some other part. It is, he says, doubtless owing to the greater prevalence of these conditions in children that diphtheria is so much more frequently contracted by them than by adults. The greater frequency of the disease and the greater mortality among children in sparsely settled districts are also, doubtless, owing to the fact that diseased tonsils and adenoid growths receive less attention in such regions than in towns and cities. As to the inoculability, contagion and dissemination of diphtheria, it is now clearly to be seen that such may take place in any manner in which the germ of the disease can be carried. It may be communicated through direct contamination of articles of food and drink, carried in wearing apparel, or derived through any source by which the bacilli can be imparted from one person to another.

After describing very clearly the characteristic appearance and behavior of the Klebs-Loeffler bacillus, the author discusses the associated microörganisms, which are frequently the cause of membranous exudates, and which so closely resemble that caused by the specific organism as to be mistaken for it. This differentiation in the characteristics of these various membranous formations of the throat was, it should be remembered, recognised by expert observers long before the *raison d'etre* was demonstrated by the microscopist, and "membranous sore throat," "diphtheroid sore throat," "croupous sore throat" and the like, were familiar terms employed to designate these respective conditions. Those germs most frequently found in this connection are mainly cocci, as diplococci, staphylococci and streptococci. When these are found independent of the bacillus, a much less serious disturbance is indicated than when associated with it. The virulence of the diphtheritic bacillus is also enhanced or diminished by the kind and degree of intermingling of these organisms.

The author then proceeds to describe a bacillus which, in its method of growth, the formation of colonies and its microscopical appearance, is absolutely identical with the Klebs-Loeffler bacillus. This bacillus is termed the "pseudo-diphtheria" bacillus. It is this bacillus which is often found in the throats of convalescent patients, but incapable of producing diphtheria, because no culture can be obtained from it. It is thus readily seen that patients may be quarantined by our health inspectors when this bacillus only is found in the throat and is mistaken for the diphtheritic bacillus. In such cases the question should invariably be settled by the culture test of the bacteriologist.

The discussion of the pathology of the disease seems to be somewhat meager in proportion to the extent of the pathological changes, both local and general, that take place. This is compensated, however, in a great measure, by the chapters on etiology, which discuss the toxic products of the diphtheria bacillus and its associates and the action of the toxines on the system, and by the chapters on prognosis, since these portions of the book describe most accurately the toxic effects of the products of the bacillus, the tox-albuminoids. In the chapter on bacteriological diagnosis we have an admirable description of the various methods employed by bacteriologists in conducting manipulations in connection with staining, the employment of the various culture-mediums, and experiments on animals.

The various subjective and objective phenomena, upon which we were accustomed to rely before the microscope came to our aid, are clearly stated in the discussion on clinical diagnosis. Notwithstanding the great facility with which we are enabled to arrive at a positive diagnosis by the aid of the microscope, we should nevertheless be so familiar with the subjective and objective phenomena as to be comparatively independent of the microscope in cases where it is not at our command.

The chapter in which a record of illustrative cases of diphtheria and its associates is given is well worthy of study. A record of twenty-four cases is given, divided into twelve classes. For each class typical cases are selected to illustrate the pathological process produced by the different organisms, some separately and others intermingled in different proportions. Together with the clinical history of each case are given colored drawings of the appearance of the throat and a microphotograph of the bacillus or bacilli present, which are especially instructive and helpful in studying the disease.

In the prognosis of diphtheria it is shown that we must take into consideration the history and surroundings of the patient, the complications of vital functions during the course of the disease, the sequelæ and the result of the bacteriological examination. Aside from the two general conditions on which reliance is placed in forming a prognosis—namely, the general condition of the patient and the virulence and extent of the disease,—we can rely very largely on the bacteriological examination.

In discussing the treatment found most effective in cases of diphtheria, Mr. Browne says :

It cannot but be gratifying to the clinician that all the advancement in scientific knowledge of the etiology of the disease which we have so gratefully recorded, has but confirmed the wisdom of the internal treatment pursued in this country" [and we might add in the United States also] "for the last forty years ; albeit it has been prescribed on what the new school may consider unscientific and empirical grounds. Iron and chlorate of potash or soda still present the sheet-anchor of our constitutional treatment of diphtheria.

The use of mercury is not regarded with great favor by the author. The most excellent results obtained from the use of calomel by some of our leading men in this country, and the powerfully antiseptic and germ-destroying properties of the bichloride solution, used locally, causes us to take a little more favorable stand in regard to mercury than does the author. We quite agree with the writer as to the advisability of administering strychnine in large doses to prevent cardiac depression, and also as to the value of alcohol in large doses, which was so strongly advocated by Chapman a few years ago.

Among the various agents used as solvents of the membrane and also as antiseptics, lactic acid is given decided preference over other agents, as lime water, perchloride of iron, carbolic acid, sulphur, sulphurous acid or sulphites, resorcine, caustics, astringents, or alkaline solutions, chloric acid and peroxide of hydrogen. He says : "We have been so well satisfied with lactic acid that we have been loth to try any other remedy. . . . . Its action appears to be limited almost solely to unhealthy tissue, promoting its disintegration by a process analogous to that of digestion." In opposition to the opinion of many writers, Mr. Browne advises, in the application of the acid with a swab, the use of sufficient friction to remove the membrane so as to reach the parts in which there is the most microbal activity.

Gargles are very justly condemned as being ineffective, not reaching the diseased part, unless used by the Von Trœlsch method, but as this requires much practice in order to avoid swallowing the solution, it is entirely impractical with children. It requires the patient to rise from the recumbent position in bed, which might be dangerous in case of weak heart ; also, it throws the muscles of the throat into irregular activity, and may, therefore, in a measure favor palatal and faucial paresis. The author advises, however, syringing the throat with a chlorine wash, or a solution of biniodide of mercury or of boric acid, as far more efficient after the removal of the membrane.

The same general plan of treatment holds good when the disease extends to or involves the nares or post-nasal space. Mr. Browne very properly urges the importance of maintaining respiration through the nares by the removal of the membrane, and also by the early incising of the membrana tympani when abscess of the middle ear occurs from the extension of the disease through the Eustachian tube. Among the operations that he wisely advocates, and which he says he has performed for seventeen years, are the removal of enlarged tonsils, of thickened and elongated uvulæ and of adenoid growths, when these parts become so swollen with disease as to be serious impediments to respiration. Such operations are condemned by many because of the exposure of a raw surface to infection. If this were so, it would still have to be admitted that such exposure is far less dangerous than interference with respiration. In the case of tonsils permeated with germs, excision leaves a much less fertile medium of incubation, and, as the author points out, if the disease is confined to the tonsils the affected part is thereby removed.

With regard to the question of croup, as differentiated from laryngo-tracheal diphtheria, we think here an unwarranted distinction is maintained. When we consider that the standard definition of croup is "a disease, generally of infants, characterised by inflammation, with fibrous exudation of the larynx and trachea," and when we remember that this fibrous exudation is, as the writer shows, produced by a germ and by the same germ that caused fibrous exudation in the fauces, we cannot believe there is sufficient reason for calling the disease *croup* when located in the larynx and trachea, and *diphtheria* when located in the fauces, even for the purpose of conforming to the customs of continental observers, as the author proposes.

There is another question that might be raised at this point. It is in regard to the author's terms "non-bacillary croup" and "non-bacillary diphtheria." As it is recognised that the appearance of the Klebs-Lœffler bacillus is the unfailing characteristic that differentiates diphtheria from other forms of mucous inflammation, would it not be more scientific to regard all cases in which the Klebs-Lœffler bacillus appears as diphtheria? We would thus be enabled to reserve the term "croupous inflammation" for those forms of mucous inflammation attended with an exudate produced by the different varieties of cocci, whether in the larynx, trachea or fauces. Clinically speaking, this distinction cannot be made without the aid of the microscope in expert hands. In the treatment of these conditions, therefore, until such distinction can be positively made microscopically, the safest rule to follow would be to regard the case as diphtheria until proved to be something else.

In the treatment of laryngo-tracheal diphtheria, Mr. Browne finds the traditional emetic of signal service, and likewise uses to advantage the Leiter coil for applying dry cold to the neck by passing ice-water through the coil. He also advocates the use of steam impregnated with pinol, and, in cases of spasm, with benzoin and chloroform. The use of vapor mixed with carbolic acid he also employs, but that obtained from slaking lime, which is so much employed and regarded as a special solvent of the membrane, he does not mention.

The two principal operative measures for relief of impending suffocation are intubation and tracheotomy. The question as to which operation should be chosen can, as the author points out, very often be decided with the laryngeal mirror. If the membrane is scanty and the swellings and obstructions are above the glottis, intubation is called for. If there is much membrane in the lower part of the larynx and in the trachea, tracheotomy is indicated. It would seem, however, that in England, as on the continent, tracheotomy is warmly advocated, and in many cases given the preference to intubation; whereas, in this country in many of the same cases the latter operation would be preferred. During his visit to this country in 1887, Mr. Browne states that he had an opportunity of seeing some of Dr. Waxham's cases, and says: "We have since had some encouraging experiences with the operation in our own practice, and we are bound to confess that many former objections, which we entertained, have been almost entirely dissipated." The superiority of intubation over tracheotomy in young children and the importance of avoiding a cutting operation are points that urgently commend it to the author; whereas, the difficulties of feeding and the great skill required in removing and cleaning the tube when obstructed, are points in favor of tracheotomy. One of the most forcible arguments against tracheotomy and one that should, we think, lead us to avoid it if possible, is the sequelæ that so often arise from the incision of the trachea. Many cases of sudden death are reported in children and in older persons who have previously undergone the operation of tracheotomy. These sudden deaths were due undoubtedly to cicatrices or growths in the interior of the trachea. It was a noticeable fact during our late war that volunteers very rarely presented themselves for examination who had undergone the operation of tracheotomy. Considering the large number of men that entered the army, and the frequency with which the operation had been done for years, this is most significant. The preventive measures to be observed with respect to the hygiene and prophylaxis of diphtheria are clearly and fully discussed. They are such as are of generally recognised importance to prevent the spread and propagation of the disease.

The value of the book is increased by the addition of a number of formulæ which the author has found of special service in the treatment of diphtheria, and they will doubtless prove equally useful to others.

As an appendix the author discusses the subject of the serum treatment of diphtheria. He details very clearly and concisely the history of the researches that led to its adoption, and also its theory of action and its method of production. Mr. Browne makes his deductions as to the value of this mode of treatment from a series of 200 cases treated in the wards of the Metropolitan Fever Hospital, in which he was able to follow the course of treatment of the cases. In 100 of these cases the serum treatment was used, and in the other 100, old and well-established methods were employed. The actual mortality in both series of cases was the same—namely, twenty-seven. In the cases under treatment the complications that arose, such as skin eruptions, affection of the joints, adenitis, renal complications—as albuminaria, anuria and nephritis—were more frequent in the cases in which the serum treatment was employed than in those treated by the classical methods. Notwithstanding the large number of reported cases in which this method has been adopted and has, according to other statistics, reduced the percentages of deaths from one-third to one-fourth of the total number of cases, it is readily seen that Mr. Browne is not favorably disposed toward the serum treatment. With his opinion some able and experienced observers in this country concur, although the great preponderance of evidence thus far is in its favor. The author regards the treatment as still on trial, and he "deprecates the acceptance of the serum treatment of diphtheria with that almost blind enthusiasm which was accorded to Koch's remedy on the high reputation of its author."

In conclusion, we cannot but congratulate Mr. Browne upon presenting the clearest and most concise brochure that has appeared upon the subject since Sir Morell Mackenzie's classic monograph, and commend it for careful study to those desirous of obtaining a full and accurate knowledge of the present relation of medical science to the etiology and treatment of diphtheria. The work is profusely and admirably illustrated by the author's own drawings, many of which have been most beautifully reproduced in color. The microphotographs of the bacilli, placed side by side with the drawings illustrating the macroscopic appearance of the fauces, are most excellent. The typographical execution of the book is all that could be desired. J. O. R.

